# Moral Distress as Subordination by Design: Silence, Containment and the Ethical Erosion of Nursing

**DOI:** 10.1111/nin.70123

**Published:** 2026-06-15

**Authors:** Alan Ramsay, Kris McBain‐Rigg, Peter Hartin

**Affiliations:** ^1^ Nursing and Midwifery, College of Healthcare Sciences James Cook University Cairns Queensland Australia

**Keywords:** advocacy, feminist ethics, moral agency, moral distress, silence, subordination by design

## Abstract

Moral distress has become one of the most prominent ethical constructs in contemporary nursing, widely used to describe nurses' experiences of constraint, frustration and ethical unease. While moral distress scholarship continues to retain important ethical and structural dimensions, the broader operationalisation of moral distress within healthcare organisations and professional discourse may influence how ethical conflict is understood and managed within healthcare organisations. This paper argues that such framings can have unintended consequences for nursing's moral authority and professional standing. Adopting a critical theoretical and conceptual synthesis, and drawing on feminist theory, Foucauldian analyses of power and contemporary nursing scholarship, this paper reconceptualises moral distress as functioning within conditions of subordination by design, understood as organisational, discursive and material arrangements that may systematically constrain nursing authority while influencing the conditions under which ethical dissent is manageable and non‐disruptive. Within this framework, this paper advances three related concepts: *moral containment*, referring to organisational responses that absorb and redirect ethical dissent; *ethical erosion*, describing the cumulative diminishment of nurses' moral agency over time, and *ethical laundering*, naming the institutional process through which ethical harm may be acknowledged yet rendered politically inert. Rather than rejecting moral distress scholarship, the paper situates moral distress within a broader architecture of organisational power, contributing to contemporary debates regarding nursing voice, professional authority and the structural conditions under which nursing ethics can be meaningfully enacted.

## 1 Introduction

Moral distress has become one of the most prominent ethical constructs in contemporary nursing, widely used to describe experiences of constraint, frustration and ethical unease. Originally articulated by Andrew Jameton (1984) as a response to institutional barriers that prevent nurses from acting on their moral judgement, the concept was grounded in an explicitly structural understanding of power, hierarchy and constraint.

Contemporary scholarship has since expanded and refined the concept in important ways. Fourie (2017), for example, argues for greater conceptual precision through distinctions such as moral‐conflict distress and moral‐uncertainty distress, while Morley et al. (2021) examine the complexity and diversity of moral distress experiences through a feminist empirical bioethics lens. These contributions retain explicit engagement with ethical conflict and moral events.

However, while contemporary moral distress scholarship often preserves structural and ethical dimensions, the broader operationalisation of moral distress within healthcare organisations may, in some contexts, redirect attention toward resilience, coping and individual adaptation. In doing so, the structural conditions that generate ethical constraint may become less visible within organisational responses to ethical conflict and may inadvertently privilege individual coping and adaptation over structural critique or institutional reform.

This shift has significant implications for nursing's moral authority. Moral distress now operates not only as a descriptor of ethical experience but also as a discourse that shapes how ethical conflict is named, expressed and managed within healthcare organisations. Ethical conflict becomes visible organisationally, but not necessarily actionable.

Understanding this tension requires a critical theoretical engagement with feminist theory, Foucauldian analyses of power and contemporary nursing scholarship. This analysis brings Dunn's (2025) account of structural subordination into dialogue with feminist scholarship on silence (Rietze et al. 2026) and emerging analyses of medical misogyny (Jackson et al. 2026), to examine how ethical dissent is shaped, expressed and constrained within contemporary healthcare.

## 2 Conceptual Clarification: Containment, Erosion and Laundering

To support conceptual clarity, it is important to distinguish between the three key terms developed in this paper. Moral containment refers to organisational strategies that absorb and manage ethical dissent without allowing it to disrupt institutional priorities. This may occur through mechanisms such as reflective practice, well‐being initiatives or ethics debriefs, which provide legitimate avenues for expression while redirecting attention away from broader structural causes.

Ethical erosion describes the cumulative impact of sustained constraint on nurses' moral agency. Over time, repeated experiences of ineffective advocacy, organisational inaction or professional risk may diminish nurses' willingness or perceived capacity to engage in ethical challenge, even when moral awareness remains intact.

Ethical laundering names these institutional outcomes. It refers to the way in which ethical concerns are acknowledged, documented and rendered visible, yet simultaneously neutralised in their political impact. Through this process, organisations may maintain the appearance of ethical responsiveness while preserving existing power structures.

Together, these concepts delineate not isolated phenomena but interconnected mechanisms through which ethical conflict is managed, contained and ultimately depoliticised. Conceptually, moral containment can be understood as the immediate organisational response to ethical dissent, ethical erosion as its cumulative professional effect and ethical laundering as the institutional outcome that stabilises this process over time.

## 3 Moral Distress and the Shift From Structure to Containment

Jameton's (1984) original account of moral distress was explicitly concerned with institutional constraint and the structural conditions that prevent nurses from acting in accordance with their moral judgement. Contemporary scholarship has since expanded and refined the concept in important ways. Fourie (2017), for example, argues for greater conceptual precision through distinctions such as moral‐conflict distress and moral‐uncertainty distress, while Morley et al. (2021) examine the complexity and diversity of moral distress experiences through a feminist empirical bioethics lens. These contributions retain explicit engagement with ethical conflict and moral events.

However, tensions remain between structural accounts of moral distress and the ways ethical conflict is often managed within organisational settings. Responses centred primarily on resilience, coping or emotional regulation may inadvertently obscure the institutional and hierarchical conditions that contribute to ethical constraint. At some point in this process, the connection between ethical suffering and power, hierarchy and exclusion may become less visible within dominant framings of moral distress. Chinn's (2016) caution regarding the ‘gradual creep’ of power‐over traditions serves as a reminder of how readily hierarchical logics can reassert themselves, even within professions dedicated to care and justice. Nursing ethics has long extended beyond reflection and resilience to include critical engagement with structural inequities and collective action, yet these dimensions are not always foregrounded in contemporary discussions of moral distress.

Revisiting Jameton's (1984) original insight clarifies what is at stake: moral distress was conceived as a response to institutional constraints rather than as a personal deficiency to be managed. Repositioning moral distress within frameworks of authority and governance restores this critical orientation.

This shift toward individualised framings of ethical conflict also resonates with broader gendered patterns within healthcare. As feminist scholars have long argued, nursing labour is culturally coded as emotional, relational and caring, all attributes that are simultaneously admired and devalued (Hochschild 2003; Tronto 2013). When ethical insight is framed primarily through emotion, it risks being interpreted not as professional judgement but as a sign of fragility. As Fricker (2007) suggests, such dynamics can amount to forms of epistemic injustice in which nurses' moral knowledge is discounted precisely because it is expressed through affective registers culturally associated with femininity.

In some organisational contexts, moral distress may function as a professionally acceptable language through which ethical discomfort can be expressed without necessarily disrupting underlying organisational arrangements. Ethical conflict may be acknowledged organisationally while responses remain oriented primarily toward individual coping, resilience or emotional adaptation rather than broader structural reform. In this sense, organisational responses may function as forms of moral containment by permitting ethical discomfort to be expressed in professionally sanctioned ways while leaving underlying governance arrangements relatively intact.

## 4 Subordination by Design: Structural Conditions of Ethical Constraint

Dunn's (2025) concept of subordination by design provides a structural account of how nursing authority may be systematically constrained within healthcare organisations. Drawing on perioperative contexts, Dunn demonstrates that subordination is not simply the product of interpersonal dynamics, but is embedded within organisational arrangements themselves. Policies, spatial configurations, procedural protocols and governance structures may simultaneously rely on nursing expertise while delimiting the scope within which that expertise can be enacted. For example, decision‐making authority may be formally located within medical or managerial hierarchies, while nurses remain responsible for coordination, safety and the ongoing management of care, creating a structural separation between responsibility and authority.

Dunn's analysis reframes phenomena such as moral distress, workplace aggression and silence as systemic outcomes rather than individual failures. While developed in perioperative settings, this insight resonates more broadly, though not uniformly, across healthcare contexts. Empirical studies of nursing work suggest that nurses frequently coordinate complex care processes while being inconsistently included in formal decision‐making forums (Allen 2014). The resulting disjuncture between responsibility and authority may be experienced as ethical constraint, particularly where nurses are accountable for patient outcomes but lack the authority to influence key decisions.

This structural configuration can be further understood through a Foucauldian lens. Foucault (1977) describes disciplinary power as operating not primarily through overt coercion, but through the organisation of spaces, practices and expectations that shape how individuals regulate their own behaviour. Within nursing, professional norms of collegiality, adaptability and emotional regulation may function as mechanisms through which expectations of compliance are internalised.

These expectations are reinforced through performance appraisal systems, professional socialisation and organisational reward structures that valorise flexibility while rendering dissent professionally risky (Allen 2014; Rushton 2024). In this sense, power operates not by suppressing ethical awareness, but by shaping the conditions under which it can be expressed, recognised and acted upon.

Within such conditions, speaking up may be encouraged rhetorically but constrained in practice. Nurses may anticipate that raising concerns will be ineffective, disruptive or carry reputational consequences, leading to forms of self‐regulation that align with organisational priorities. In this sense, subordination by design does not eliminate ethical awareness, but shapes the conditions under which it can be expressed and acted upon.

## 5 Silence, Voice and the Ethics of Speaking

Recent scholarship has paid increasing attention to silence as an ethical and political phenomenon. Rietze et al. (2026) demonstrate that silence in nursing is rarely the absence of knowledge, concern or moral reasoning. Rather, silence often represents a strategic and ethically rational response to organisational power, risk and disciplinary cultures that punish dissent while rhetorically valorising speaking up.

Dunn (2025) and Rietze et al. (2026) both challenge the idea that voice or silence is simply a personal choice or moral failing. Nurses may know what needs to be said, but accurately anticipate that speaking will lead to marginalisation, reputational harm or futility (Rietze et al. 2026). Silence, in this sense, can function as a survival strategy within hierarchies that render nursing knowledge visible yet contestable. When moral distress is positioned as the primary ethical response to constraint, it may unintentionally reinforce this dynamic. Nurses may be encouraged to name their distress, reflect upon it and seek support, yet the organisational conditions that render speech ineffective can remain intact.

Ethical conflict may be acknowledged organisationally while responses remain oriented primarily toward individual coping or resilience rather than broader structural reform. In this way, organisational responses can function as forms of moral containment. Such responses may offer support but can also risk privileging forms of expression that align with organisational expectations while marginalising those whose distress, burnout or dissent more directly expose structural tensions.

## 6 Medical Misogyny and the Gendered Regulation of Ethics

The containment of nursing ethics cannot be fully understood without reference to gender. Jackson et al. (2026) identify misogyny in healthcare not as isolated prejudice or interpersonal hostility, but as a structural and discursive force that regulates credibility, authority and whose knowledge is recognised as legitimate.

Within clinical hierarchies, misogyny often operates less through overt exclusion than through everyday institutional practices that reward compliance, emotional restraint and neutrality, while casting ethical resistance as disruptive, inappropriate or professionally suspect. Within this context, nursing's ethical voice may be doubly constrained: first by professional hierarchies that privilege medical authority, and second by gendered expectations that position nurses as supportive, accommodating and non‐confrontational (Jackson et al. 2026). These expectations are not merely incidental but constitutive of nursing's professional identity, shaping how ethical concerns are expressed and received (Hochschild 2003).

When nurses discuss ethical issues, they may be dismissed as overly emotional, overreactive or unprofessional. These judgements draw on entrenched cultural associations between femininity, affect and diminished epistemic credibility, which Fricker (2007) describes as testimonial injustice. Crucially, these gendered dynamics intersect with contemporary moral distress discourse in ways that may further constrain nursing ethics. As Fourie (2017) cautions, when ethical conflict is reframed as an internal emotional struggle, responsibility may become subtly relocated from organisational structures to individual clinicians.

In some contexts, moral distress discourse may unintentionally align with broader gendered expectations surrounding emotional expression and professional comportment within healthcare hierarchies. Distress becomes evidence of caring rather than of constraint; endurance is celebrated while critique is softened.

## 7 Ethical Laundering: The Mutual Reinforcement of Moral Containment and Ethical Erosion

Taken together, these analyses suggest that moral distress can be understood as functioning within a broader system of ethical regulation. Moral containment refers to organisational processes that permit ethical discomfort to be expressed in sanctioned forms that do not threaten institutional priorities. Wellness initiatives, resilience training and reflective spaces may provide authorised outlets for distress while leaving underlying hierarchies and decision‐making structures intact.

Over time, repeated exposure to such containment can contribute to ethical erosion. When nurses learn that speaking up carries personal risk, yields little change or is quietly redirected into self‐management, moral engagement may gradually be withdrawn. Moral sensitivity persists, but moral agency may diminish. Within this interpretive framework, ethical laundering is proposed as a conceptual lens through which the cumulative organisational management of ethical concern may be understood. Through ethical laundering, organisational harm may be rendered visible but politically inert, stripped of its critical force and returned to nurses as something to endure, regulate and survive rather than contest or transform.

Importantly, ethical laundering is not presented here as an episodic phenomenon but as a cumulative and collective one. It may shape professional identity, socialise new nurses into silence and reinforce the perception that nursing ethics reside primarily in emotion and endurance rather than in epistemic authority or political judgement.

## 8 Reclaiming Nursing's Moral Authority

New strategies must extend beyond resilience‐based interventions. As Delgado et al. (2020) note, responses focused primarily on individual adaptation may leave the underlying structural conditions of ethical constraint unchanged. Instead, policies should prioritise enhanced authority, meaningful participation in governance and protections against political decisions that threaten the profession's autonomy.

As Morley and Sankary (2024) point out, moral agency is dependent on the structural conditions under which nurses practise. Without influence in policy decisions, nurses may remain susceptible to forms of ethical erosion that moral distress often signals. Ensuring nursing representation in decisions that shape classification, regulation and education is likely to be essential to resisting de‐professionalisation and sustaining moral integrity across healthcare systems.

Reframing moral distress as structurally produced opens new avenues for ethical action. Rather than asking how nurses might better endure or regulate distress, a critical ethical approach asks how organisations might redistribute authority, legitimise dissent and embed nursing voice meaningfully within governance structures. Dunn's (2025) call to reposition nurses as architects rather than assistants of care offers a concrete starting point. Moral distress can therefore be understood not merely as evidence of individual inadequacy, but as a signal of broader organisational and structural tensions requiring collective and institutional attention.

## 9 Conclusion

Moral distress remains an important ethical construct within nursing scholarship because it provides language through which experiences of institutional constraint and ethical conflict can be articulated. This paper has argued, however, that the organisational and professional operationalisation of moral distress may, in some contexts, contribute to forms of ethical individualisation that risk obscuring the structural conditions under which such distress emerges.

By bringing contemporary moral distress scholarship into dialogue with feminist theory, Foucauldian analyses of power, and Dunn's (2025) concept of subordination by design, this paper advances a structural and political interpretation of ethical constraint within nursing. In doing so, it proposes the concept of ethical laundering as a way of understanding how ethical concerns may become acknowledged institutionally while simultaneously neutralised in their disruptive or political potential. Rather than rejecting moral distress scholarship, this paper seeks to extend ongoing discussions regarding nursing voice, organisational power and the conditions necessary for sustaining moral agency within contemporary healthcare systems.

## Funding

The authors have nothing to report.

## Ethics Statement

The authors have nothing to report.

## Conflicts of Interest

The authors declare no conflicts of interest.

## Data Availability

Data sharing not applicable to this article as no datasets were generated or analysed during the current study.
